# Therapeutic Drug Monitoring of Low Methotrexate Doses for Drug Exposure and Adherence Assessment—Pre-Analytical Variables, Bioanalytical Issues, and Current Clinical Applications

**DOI:** 10.3390/ijms252413430

**Published:** 2024-12-14

**Authors:** Arkadiusz Kocur, Aleksandra Mikulska, Mateusz Moczulski, Tomasz Pawiński

**Affiliations:** 1 Department of Drug Chemistry, Pharmaceutical and Biomedical Analysis, Faculty of Pharmacy, Medical University of Warsaw, Banacha 1, 02-097 Warsaw, Poland; 2 Student’s Scientific Association, Drug, Department of Drug Chemistry, Pharmaceutical and Biomedical Analysis, Faculty of Pharmacy, Medical University of Warsaw, Banacha 1, 02-097 Warsaw, Poland

**Keywords:** methotrexate, methotrexate polyglutamate, LDMTX, therapeutic drug monitoring, adherence

## Abstract

Methotrexate (MTX) is an antifolic agent used in the first line of anti-inflammatory disease treatment and some oncologic issues. The metabolism of MTX is rapid, and the MTX concentration in the blood is not significant 24 h after administration. Unlike this, methotrexate polyglutamates (MTXPGs) can be informative biomarkers of drug exposure. It is widely concluded that MTXPG retention in red blood cells (RBCs) is related to appropriate efficacy, drug exposure, and toxicity during treatment. Therefore, the mentioned biomarker may be appropriately used for the PK/PD monitoring of low-dose MTX (LDMTX) treatment. The presented review study aimed to review published studies about MTX determination in clinical practice, including pre-analytical variability, bioanalytical considerations, and clinical applications of the methods for pharmacotherapy supporting target populations. In total, 14 papers from the field of bioanalytics have been included in the main review. For each phase of an analytical process, the best practises and main findings were defined as guidelines for proper analytical method optimisation, validation, and standard operation procedure implementation in clinical practice. The presented study is the first comprehensive review of MTX and its methods of metabolite determination to account for pre-analytical, analytical, and post-analytical phases concerning the TDM process.

## 1. Introduction—Basic Information About Methotrexate (MTX)

**Figure 1:**
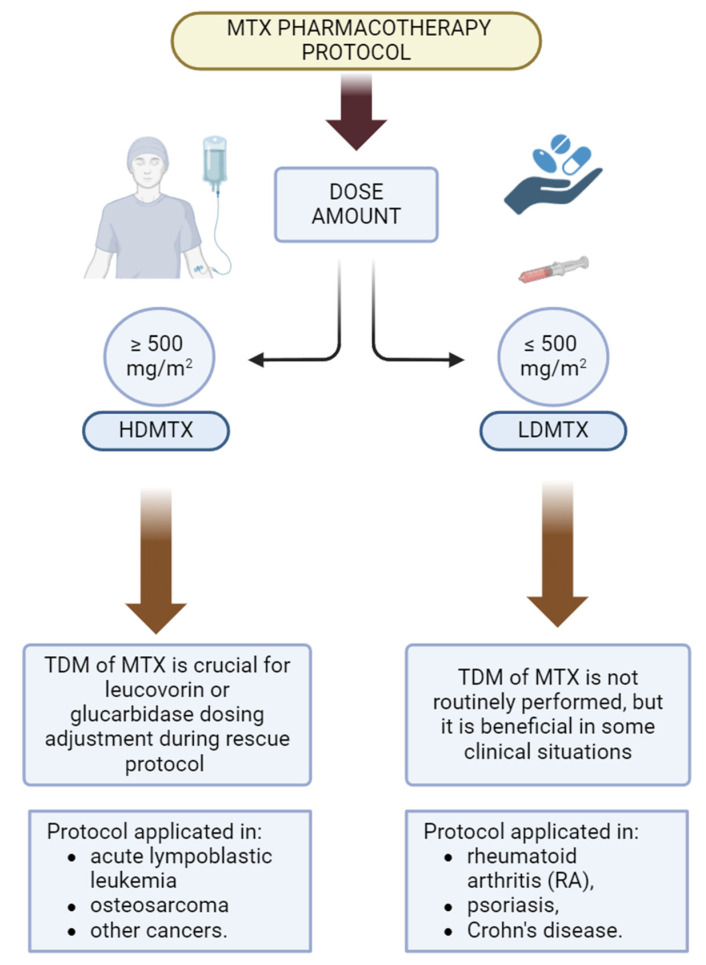
Methotrexate is administered clinically under two protocols, regarding dose amount—HDMTX (high dosing of methotrexate) and LDMTX (low dosing of methotrexate). Abbreviations: TDM—therapeutic drug monitoring. The figure was created using bioRender.com under publishing rights.

Methotrexate (MTX; aminopterin; (2*S*)-2-[[4-[(2,4-diaminopteridin-6-yl)methyl-methylamino]benzoyl]amino]pentanedioic acid) is considered one of the main chemotherapeutic agents with antifolic activity used in clinical practice for over 60 years [1]. The World Health Organization lists MTX as an essential drug in treating various diseases [2]. In its current clinical status, MTX is applied in oncology in high-dose protocol (HDMTX) and the treatment of anti-inflammatory diseases in low-dose protocols (LDMTX) [3,4]. Primary indications, routes of administration, and threshold doses are shown in Figure 1 [3,4,5]. MTX was initially developed for leukaemia treatment, and it was used commonly in rheumatoid arthritis (RA) in the 1980s. However, the pharmacotherapy schemes in rheumatology were updated after the introduction of biological drugs. On the other hand, MTX is considered a first-line treatment in RA [6]. Autoimmune diseases, including RA, affect more than 5% of the adult population globally, including more than 1% with RA [6,7,8]. Notably, MTX is recognised as a conventional synthetic disease-modifying antirheumatic drug (csDMARD), which means that it relieves inflammation, pain, and rheumatoid arthritis (RA) disease progression [6,7,8]. The one of the main problems associated with LDMTX is the variable clinical response for pharmacotherapy. In this case, MTX is highly variable, ranging from 40 to 60%, depending on the studies available in the literature [9,10,11,12,13,14].

**Figure 2:**
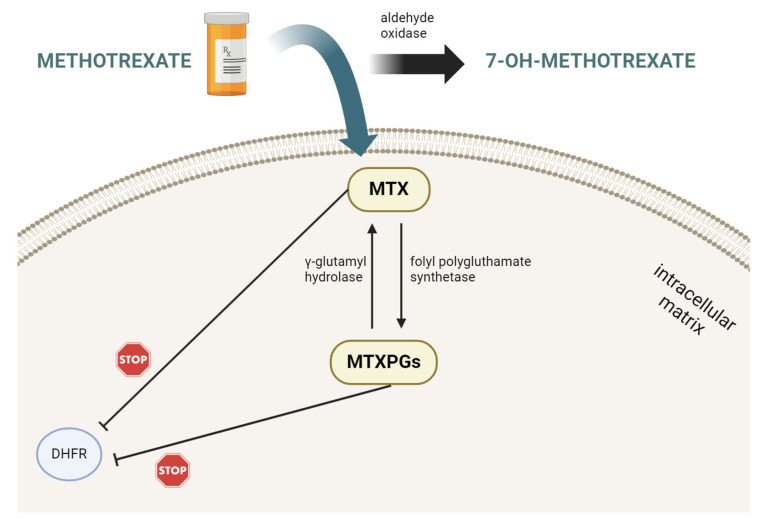
Simplified mechanism of MTX action. Methotrexate is a potent conversion inhibitor from 5,10-methylenetetrahydrofolate to 5-methyltetrahydrofolate by methylenetetrahydrofolate reductase. The main metabolites of MTX are 7-hydroxy-methotrexate and methotrexate polyglutamates. Abbreviations: MTX—methotrexate, DHFR—dihydrofolate reductase, and MTXPGs—methotrexate polyglutamate. The figure was created using bioRender.com under publishing rights.

The mechanism of MTX action assumed interaction with several critical biochemical pathways. After penetration into the cell–matrix, MTX is polyglutamated, and the products of this reaction inhibit the dihydrofolate reductase (DHFR) [3]. Enzyme folylpolyglutamate synthetase (FPGS) catalyses the addition of up to seven glutamate residues to MTX intracellularly within 24 hours after administration (Figure 2) [3]. The glutamates are sequentially linked with MTX, caused by [MTXPG]_n_, where *n* = 2–7. MTXPGs (methotrexate polyglutamates) bind to DHFR with an affinity 1000 times higher than folic acid. MTX interacted with nucleotide synthesis and DNA, RNA, and the immunological system by modulating cytokine synthesis. Additionally, MTX may initiate the programmed cell death of activated lymphocytes T cells [1,2,3].

**Figure 3:**
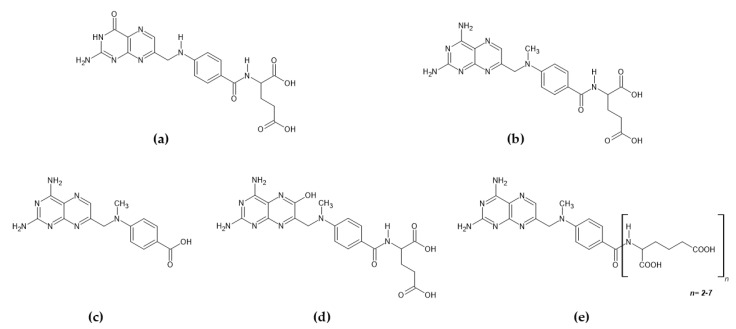
Chemical structures of folic acid (**a**), methotrexate (**b**), DAMPA (2,4-diamino-N-10-metylpteroic acid (**c**), 7-hydroxymethotrexate (**d**), and MTXPGs (methotrexate polyglutamates) (**e**).

Chemically, MTX is a structural analogue of folic acid. The chemical structure of the mentioned agents is provided in Figure 3. Compounds c–e are metabolites of MTX.

Some studies concluded that the LADME of MTX drastically differs in population, especially during the HDMTX protocol [2,3,4]. As MTX is the first-choice agent in RA treatment, differences in drug response among patient groups are regarded by different factors, including pharmacogenetic factors, patient factors (age, renal function, co-morbidities), and concomitant treatment with other drugs [2]. The basic pharmacokinetics parameters regarding MTX and their metabolites are summarised in Table 1 [1,3,4,15].

Low doses of methotrexate (LDMTX) are used for rheumatoid arthritis (RA), Crohn’s disease, and psoriasis treatment [6,7]. The oral bioavailability of low doses of MTX (<30 mg/m^2^) is relatively high (~70–90%), but decreases with increases in dose amount. Methotrexate is excreted mainly via the renal route, and the approximate drug clearance may be estimated using the creatinine clearance value (CL_CR_ × 1.60) [3]. Parent drugs (MTX) and their metabolites are detected in whole blood, with poor pharmacological activity of 7-OH-MTX and DAMPA [3,4]. The amount of excreted 7-OH-MTX is highly variable, ranging from 1% to 20%, while DAMPA levels are not fully recognised [3]. However, the 7-OH-MTX is characterised by ~0.5–1% of the initial pharmacological activity of MTX, the 7-OH-MTX solubility in water is lower than acidic MTX, and its crystals cause renal damage, such as nephrotoxicity [3]. Therefore, an appropriate fluid supply, including sodium carbonate, is necessary, characteristic of HDMTX protocol. After oral administration, around 10% of MTX is metabolised in the small intestine to 7-OH-MTX [16].

According to the pharmacokinetics of MTX, the most suitable model is a two-phase (two-compartment) model for appropriate dosing adjustment [17]. In practice, for MTX concentrations higher than 0.50 µM, the half-life time is estimated as 3 h, while that for amounts below the mentioned threshold is 10 h. Routinely, the time points are crucial in the TDM of HDMTX. Namely, after 24–36 h, the determinate MTX concentration is in steady state, and after 48 h, it serves as a surrogate of MTX elimination dynamics, which determines leucovorin dosing. The MTX concentration is obtained in a sample collected after infusion finishes after 60 h, and the length of rescue leucovorin administered is determined. The pharmacokinetic parameter the most appropriate for the clinical TDM of MTX is the concentration at steady state (C_ss_) [5]. In LDMTX protocol, the weekly dose of MTX is eliminated within 24 h, starting from drug administration [3,4,5]. MTX is used in relatively high doses, expressed in mg or gram per body surface area when MTX concentration is expressed as mg/L or molar concentrations. For unit recalculations, the MTX concentration in mg/L may be divided to 0.454 to obtain the molar concentration (in 10^−6^ M). The 2.20 factor might be used for MTX conversion from mg/L to µmol/L [3,4].

The toxic effects of MTX may include vomiting, nausea, myelosuppression, mucosal ulceration, renal or hepatic dysfunction, rash, and other side effects. TDM of MTX is also beneficial due to other PK interactions with some drugs, such as non-steroidal anti-inflammatory drugs (NSAIDs) [3,4,18]. This interaction should be monitored clinically by ALT/AST activity determination to avoid hepatotoxicity [18].

Despite the above, pharmacotherapy with MTX is still tricky, despite its well-established position in clinical treatment. Therefore, TDM is beneficial in dose adjustment, the prevention of toxic side effects, and toxicity minimisation. The main aim of methotrexate level monitoring is the prevention of acute toxicity and dosing adjustment. On the other hand, TDM may be beneficial in adherence monitoring, especially during low doses of MTX pharmacotherapy [9,10,11,12,13,14,19,20,21].

Appropriate analytical techniques for drug concentration determination are crucial in the actual clinical practice of TDM. For many agents, the chromatographic techniques are essential and considered a reference point [21]. The most frequently used analytical methods for the quantification of MTX (and metabolites) are chromatographic applications and immunoassays. Additionally, spectrophotometric and electrophoretic methods are used in biomedical, pharmaceutical, and environmental analysis of MTX levels [22,23]. Historically, methods of MTX determination were recognised as RIAs (radioimmunoassays), DHRF enzyme-reducing tests, and chromatographic assays with ultraviolet (UV) or fluorescence (FLD) detection [19,22,23].

MTX is highly distributed into erythrocytes, with a relatively high blood-to-plasma ratio (more than 1). Therefore, measuring MTX as intracellular MTXPGs seems to be an appropriate approach for treatment monitoring, mainly because the MTXPGs are responsible for the anti-inflammatory activity of methotrexate action. However, the LDMTX protocols are not monitored routinely, but, on the other hand, MTX is considered a drug with a relatively narrow therapeutic range [3,4,24]. Additionally, appropriate drug intake assumed appropriate therapy. MTX levels are low, but MTXPGs are considered surrogates of treatment efficacy according to RBC turnaround life-time [25]. MTXPGs are formed in the cell matrix by folylpolyglutamate synthetase and are recognised as an active form of MTX, which causes the intended treatment effect. Due to the enzyme being widely present in erythrocytes (red blood cells, RBCs), the appropriate matrix for MTXPG determinations is the RBC compartment [22,23,25,26].

The presented study aimed to review published studies about MTX determination in clinical practice, including pre-analytical variability, bioanalytical considerations, and clinical applications of the methods for pharmacotherapy supporting target populations. In the following sections in paragraph 3, the chromatographic methods are reviewed according to clinical application, methodology, and potential issues. Despite the commercial application of immunochemical methods, these have been discussed in paragraph 4. Immunoenzymatical methods, fluorescence, and electrochemical methods have historically been used to monitor MTX-PGs. Only in LC-MS/MS methodology can MTX and MTX-PGs be determined simultaneously. There are several contributions to MTX (and metabolite) determination using chromatographic and immunochemical techniques. Since the LC-MS/MS methods are seen as the gold standard in MTX determination regarding lower and higher doses, the methodologies based on that technique have been included in the proper part of this review. There are several analytical applications of MTXPGs determination, from enzymatic to chromatographic determination using LC-MS/MS platforms in microsamples, such as DBS and VAMS [19,20,21,22,23]. In the next paragraph, special attention is paid to analytical methodologies helpful for appropriate therapeutic and adherence monitoring of patients under LDMTX treatment.

To the best of our knowledge, the presented research is the first comprehensive review of MTX (and its metabolite) determination methods to account for pre-analytical, analytical, and post-analytical (clinical) phases concerning the TDM process.

## 2. Materials and Methodology

The studies were identified for appropriate review by searching the PubMed database. For this study, analytical and clinical pharmacokinetics papers dedicated to MTX bioanalysis have been included in this review. The following scheme describes the stages of article selection to the final review. The pharmacokinetics of MTX have been widely described in the literature; therefore, in the present study, investigations on TDM were included only for accurate and solid discussion. In August 2024, the scientific literature databases, such as PubMed, Medline, and Scopus were comprehensively researched using a combination of drug names: ‘methotrexate’, ‘7-OH-MTX’, ‘DAMPA’, and ‘methotrexate polyglutamate’, with ‘adherence’, ‘LDMTX’, ‘TDM’, ‘analytical determination’ and ‘chromatography’.

**Figure 4:**
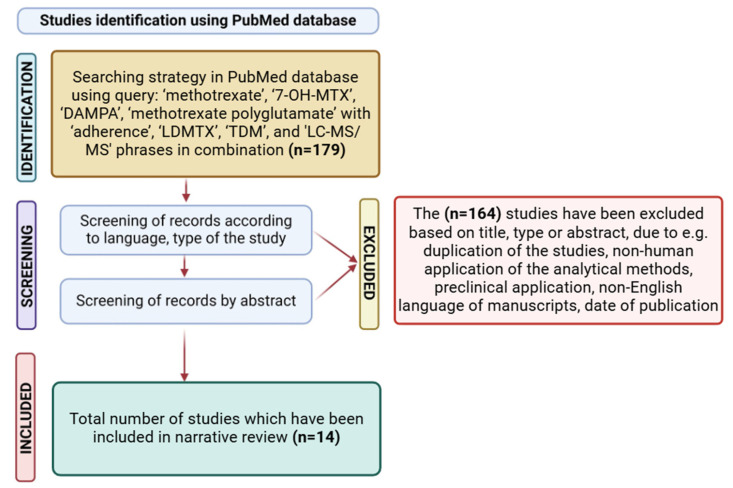
Research flowchart of study identification to narrative review.

Additionally, the bibliographies of the selected manuscripts were evaluated manually according to their potential compatibility with the aim of the presented review study. Letters to the editor, reviews, case reports, and preclinical animal studies were excluded. Another criterion for inclusion was the English language of the manuscript text. Inappropriate (e.g., studies on analyte determination in pharmaceutical forms) and duplicate studies were discarded after evaluating their abstracts, titles, or keywords. The search was adjusted to 2010–2024 for a more comprehensive review. Finally, 13 records were yielded in the presented review of analytical methodologies and for clinical evaluation of the treatment. The presented flowchart and searching methodology have been adapted from PRISMA guidelines about the systematic review to clarify the literature research process (Figure 4) [27].

## 3. Immunochemical Applications for MTX Determination

The immunochemical applications of MTX determination are well known, but their main limitation is their limited sensitivity according to LDMTX protocols. IAs are very attractive for laboratories due to their limited sample preparation and rapid analysis turnaround time [28,29,30,31]. Additionally, immunoassays (IAs) are limited due to cross-reactivity with MTX metabolites and folates. The LLOQ of IA is around 20 nM, primarily due to interference with DAMPA [28,29,30]. Another serious interference in IAs is caused by foliates. Therefore, LC-MS/MS platforms are considered a highly selective gold standard for MTX and its metabolite concentration measurement. Exemplary, the ARK^TM^ test for MTX determination represented a 100% cross-reactivity with DAMPA, lower cross-reactivity with 7-OH-MTX (≤0.07%), and limited with foliates (≤0.01%) [29,31]. The issues in fluorescence polarisation immunoassay (FPIA) for MTX are like the ARK^TM^ methodology. FPIA is characterised by cross-reactivity with DAMPA, some antibodies, an established level of cross-reactivity with 7-OH-MTX, and folates [28,29,30,31]. In some cases, except LDMTX protocols, the sample’s dilution may be necessary due to the wide range of calibration curves. Typically, the MTX-PGs are not significantly present in plasma fractions. Still, as previously reported, MTXPGs are determined in IAS as MTX, especially in the presence of haemolysis in serum or plasma [30].

## 4. Chromatographic Application—From Pre-Analytical Variables to Clinical Issues

All analytical processes may be divided into a few parts, according to sample processing and the quality of results obtained. The pre-analytical phase is related to the sample (and its type, i.e., matrix) collection process and the analytes’ stability in the matrix [29]. The analytical step refers to sample pretreatment and the primary determination of the target compounds. The post-analytical step regards results obtained, their interpretation, and their clinical implementation. The following paragraphs describe the characteristics of the mentioned phases related to MTX and its metabolite testing based on the assembled literature.

### 4.1. Pre-Analytical Phase—Sampling Process and Sample Stability

MTX and their metabolites may be determined in various matrices, including whole blood, plasma, serum, RBC (red blood cell) pellets, PBMCs (peripheral blood mononuclear cells), CSF (cerebrospinal fluid), saliva, and urine [23]. Alternative sampling strategies such as DBS (dried blood spot), DPS (dried plasma spot), and VAMS (volumetric absorptive microsampling) devices have also been used [22,23]. Therefore, various instruments for sample collecting should be used, i.e., appropriate test tube colour labels or microsampling devices. Recommended anticoagulants for obtained plasma include heparin sodium or lithium (green cap), K_2_-lub K_3_-EDTA (lavender cap), and sodium citrate (blue cap). The appropriate tubes come with or without a clot activator for serum collection. The tubes with gel are not suitable due to the slow absorption of MTX in the separator [22,23,24,25,26,27,28,29,30]. If the sample is incidentally collected into the tube with a gel separator, the serum should be centrifuged and transferred to another vial within 2 h of sample collection. Notably, mild haemolysis and lipemia do not interfere with the determination of freshly collected samples [30]. The long-term storage of haemolysed serum may cause back conversion of metabolites to free MTX [30].

The sample for MTX-PG determination should be collected as whole blood. After centrifugation, the plasma must be removed. The RBC pellet should be washed using phosphate-buffer saline or water a few times. The washed cell pellets may be frozen, preferably at −80 °C. Some erythrocytes may break during centrifugation, sample storage, or vigorous shaking (even without visible haemolysis). Therefore, MTXPGs may be abnormally presented in this fraction and obscure the results [30].

Due to MTX’s light sensitivity, stock solutions, working solutions, calibrators, and biological samples should also be protected from light. As a best practice, during more extended storage, the test tube containing the sample should be protected from light by being transferred to an amber tube or coated with aluminium foil.

Table 2 compares the biological matrix type and amount used for each assessed study.

The best practises according to this step are the following [22,23,30]:

(1) MTX and its metabolites are light-sensitive—avoid exposing the matrix sample to direct light.
(2) It is suggested that RBC samples are stable, according to MTXPGs concentration 6 months at −80 °C.
(3) After whole blood collection, plasma/serum fractions should be centrifuged within 2 h and transferred to an amber vial.

### 4.2. Sample Pretreatment as an Integrated Part of the Analytical Process

Sample pretreatment for chromatographic analysis is a crucial part of the analytical process. Methodology optimisation for analyte extraction provides a higher recovery rate when it is made adjustable to the applicated detection method. Typically used extraction methods include protein precipitation (PPT), liquid–liquid extraction (LLE), and solid-phase extraction (SPE). Sometimes, additional steps are implemented for better process efficiency and sample purification [22,23].

PPT is the most straightforward methodology of MTX extraction and sample purification. As precipitation reagents, organic solvents soluble in water may be applied. In some cases, nonorganic heavy metal salts are used in mixtures with acetonitrile or methanol. Perchloric acid and trifluoroacetic acid (TCA) have been used successfully in some cases, but on the other hand, both may interfere during analysis with MS detection. Due to the chemical properties of MTX, the presented approach seems to be complicated. Potentially, precipitation reagent acidification may improve the recovery rate, but approaches with the non-modified PPT process were also published [32].

LLE is another simple extraction technique that provides satisfactory purification and extraction. In the case of MTX, the LLE may be performed with or without aqueous phase acidification [22,23]. If the MTXPGs are determined, the described method is inefficient without RBS lysis. Therefore, this type of extraction should be preceded with erythrocyte lysis using low temperatures (freezing) or strong acids (TFA, perchloric acid). Only one method involved LLE and ethyl acetoacetate followed by lysis with cold temperatures and TFA [21,33].

The best analyte extraction and sample purification method is the SPE technique [29]. It allows for analyte extraction from highly complex matrices. The possibility of sample concentration is strictly beneficial for this type of extraction. On the other hand, SPE is a costly and highly laborious extraction technique [29]. In the literature researched for this review, only one study described applying the SPE technique for sample purification and analyte extraction (MTXPGs) after protein precipitation [34]. In the mentioned study, an appropriate analyte extraction methodology was highly criticised because a small amount of capillary blood was collected using a VAMS Mitra^TM^ device. It should be noted that a proper sample extraction process is required for dried microsampling methods to ensure sufficient recovery [21,22,29,33].

Implementing the total MTX approach (sum of MTX and MTXPGs) requires checking hydrolysis correctness (enzymatic conversion). In the suggested approach, the MTXPGs are back converted in vitro/ex vivo to free MTX in the RBC sample. In parallel, the MTX concentration is detected in the whole blood sample before conversion [16]. As a result, the total PG concentration is expressed using simple subtraction. Mo et al. assessed the conversion rate using the native solutions of corresponding MTXPGs and performed an enzymatic reaction in developed conditions [16]. The enzymatic conversion percentage rate was higher than 92%. Therefore, ensuring appropriate conditions during additional sample treatment steps is highly important, i.e., ascorbic acid application [21,22,23,33]. Studies involving the quantification of MTX and the sum of MTX and MTXPGs performed hydrolysis in in vitro conditions, i.e., for 3 h with 200–500 mM of ascorbic acid solution or mercaptoethanol. The acidification of the sample relatively accelerated the conversion from PGs to free MTX [21,22,23].

An interesting approach to MTX determination has been introduced by Daraghmeh et al. [16]. In the mentioned study, the authors validated a multi-matrix analytical method for MTXPG determination in whole blood, RBS, leukocytes, and VAMS samples. The last approach is especially beneficial due to the possibility of involving only small amounts of blood cand the self-sampling home-based remote monitoring of MTX, 7-OH-MTX, and MTXPG levels [16]. On the other hand, the small portion of matrix required the application of highly sensitive methods, such as LC-MS/MS platform and vigorous analyte extraction methods. The stability assessment is also crucial—the benefit of remote drug monitoring is only present when the stability of the analytes is satisfactory in various (potential) conditions, i.e., during shipment [35,36].

The comparison extraction protocol for each assessed study is provided in Table 2.

Best practises according to this step include the following [21,22,23,29,33]:

(1) PPT is suitable for MTX and its derivatives extraction from biological matrices, but modifications (i.e., acidification) may be required depending on the sensitivity of the analytical platform.
(2) LLE is beneficial for additional sample purification or concentration measurement after PPT. Notably, neutral or acidic conditions suit MTX extraction relatively satisfactorily.
(3) SPE is laborious and highly expensive for routine application in MTX/MTXPG determination, but it seems to be the best method for sample purification, concentration, and appropriate analyte extraction.
(4) Microsampling approaches are beneficial in ‘special’ populations, i.e., children and adolescents. Measuring each detectable MTXPG (*n* = 2–5) in RA treatment is achievable and reliable.
(5) Different approaches to MTX and MTXPGs measurement have been established in this paper:
  - Individual assaying each long-chain MTXPG concentration.
  - Determining MTX and the sum of MTX and MTXPGs. The result is expressed as total MTXPG concentration via subtraction.
  - Determining MTX and short-chain MTXPGs is not suitable due to the quick elimination of MTX from serum.

### 4.3. Review of Analytical Applications—Searching for the Ideal Solution

The presented review is focused on analytical methods based on MS/MS detection. MS is a highly sensitive and selective detector, which requires appropriate conditions to be established during method development. In most cases, MTX and its derivatives are detected with satisfactory intensity in positive ionisation mode, with single proton ionisation. Wu et al. presented another approach, where, for MTXPGs, the parent ion was double-charged, which increased the sensitivity of the detection due to higher signal intensity [35]. The application of SIL-IS (stable isotope labelled internal standard) is recommended by European Medicines Agency guidelines about bioanalytical method validation [37]. SIL-IS compensates matrix effects with high efficiency, but the costs of these standards are significantly higher than structural related or unrelated compounds. Based on the studies reviewed, the interest in SIL-IS for MTX and its metabolite determination is relatively high, which ensures the analytical method’s appreciable recovery and sensitivity [35,37].

C_18_ particles are the most suitable for TMX and the determination of its derivatives. There are a few applications with other chromatographic column applications, namely PFP (pentafluorophenyl phase) and HILIC (Hydrophilic Interaction Liquid Chromatography) [16,37]. As a mobile phase, the gradient mode of water and organic solvents has been used with the addition of modifiers, such as sodium bicarbonate and ammonium acetate. Isocratic mode mobile phase flow has also been applied [18,33].

A comparison of analytical aspects for each reviewed study is provided in Table 2.

Best practises according to this step involve the following [16,17,18,25,32,33,34,35,36,37,38,39,40,41]:

(1) LC-MS/MS platforms are the gold standard for simultaneously determining MTX and its metabolites in various biological matrices.
(2) The most suitable chromatographic column is C_18_ particles in ordinary conditions with gradient mode mobile phase flow. Due to MTX’s chemical nature, adding sodium bicarbonate to the mobile phase may be beneficial.
(3) Most applications in the literature involved positively charged multiple reactions monitoring detections of MTX and its derivatives in electrospray ionisation mode.

**Table 2:** Analytical protocol summary for MTX, 7-OH-MTX, and MTXPG determination using LC-MS/MS technique.

Analytes (Method)	Biological Matrix		Internal Standard	Sample Pretreatment	Chromatography		Parameters of Mass Detection	Validation Parameters				Stability of Analytes in Collected Sample	Reference
					Mobile Phase	Column		LOD	LLOQ	Linearity	Recovery		
**MTX;** **MTXPGs (total)**	whole blood (400 or 100 µL)		doxofylline	freeze-induced lysis of RBC, PPT combined with LLE (50% trifluoroacetic acid and extraction was performed using ethyl acetoacetate)	ACN:H_2_O (30:70, *v*/*v*) with 1% FA, 20 mM AF; isocratic; flow: 0.2 mL/min	XB-C18 3 µm; 2.1 × 100 mm	(ESI-LC-MS/MS) 455.2 → 308.2 for MTX 267.2 → 181.2 for IS	0.5 ng/mL	1 ng/mL	1–100 ng/mL	29.30–37.80%	−80 °C (1 month); 2 freeze–thaw; RT (4 h)	[33]
**MTX**	serum (30 µL)		pterin	PPT with methanol	1% acetic acid/ACN (88:12, *v*/*v*); isocratic; flow: 0.5 mL/min;	Luna C_18_ (2) 3 µm; 100 × 4.6 mm	(ESI-LC-MS/MS) 455.2 → 308.1 for MTX 164.10 → 164.10 for IS	3 nM	10 nM	10–1000 nM	100.40%	nd.	[18]
**MTXPGs** (*n* = 1–5)	RBC pellet (200 µL)		^13^C_5_,^15^N-MTXPGs (*n* = 1–5)	lysis of RBC, cold-induced PPT with 16% perchloric acid	A: 10 mM NH_4_HCO_3_ (pH = 10) B. MeOH; gradient; flow: 0.3 mL/min;	Acquity BEH C_18_ 1.7 µm; 100 × 2.1 mm	(ESI-LC-MS/MS) MTX and (IS): (1) 455.2 (461.2) → 308.2 (2) 584.4 (590.4) → 308.2 (3) 713. 4 (719.4) → 308.2 (4) 842.4 (848.4) → 308.2 (5) 971.6 (977.6) → 308.2	nd.	1 nM	0.975–1000 nM	54–98% (corrected with IS 89–108%)	−80 °C (3 m)	[25]
**MTX;** **7-OH-MTX**	urine (50 µL)		D_3_-MTX	PPT with ACN	A: water with 0.1% FA B: ACN with 0.1% FA; gradient; flow: 0.3 mL/min	Hypersil GOLD C_18_ 1.9 µm; 100 × 2.1 mm	(ESI-LC-MS/MS) MTX 455.1 →308.1 IS 458.1 → 311.1 7-OH-MTX 471.1 →324.1	nd.	MTX 2.5 nM 7-OH-MTX 10 nM	5–1000 nM	MTX (103.64–129.74) 7-OH-MTX (67.08–93.26)	significant loss of 7-OH-MTX within 72 h of storage at RT in contrast to storage at −80 °C for 168 h	[32]
**MTXPGs** (*n* = 1–7)	RBC pellet (250 µL) PBMC pellet (250 µL) Plasma (250 µL) VAMS (20 µL) Whole blood (20 µL)		^13^C_5_,^15^N-MTXPGs (1–7)	SPE with Strata-X-A Strong cartridges following PPT with 30% perchloric acid; VAMS extraction with MeOH and continuation of extraction	A: 75% ACN in H_2_O (10 mM NH_4_HCO_3_) B: H_2_O (10 mM NH_4_HCO_3_); gradient; flow: 0.4 mL/min	ZIC-pHILIC 5 µm; 100 × 4.6 mm	(ESI-LC-MS/MS) MRM (IS): (1) 455.2 (461.2) → 308.1 (2) 584.4 (590.4) → 308.1 (3) 713. 4 (719.4) → 308.1 (4) 842.4 (848.4) → 308.1 (5) 971.6 (977.6) → 308.1 (6) 550.3 (553.5) → 308.1 (7) 615.0 (618.3) → 308.1	nd.	MTXPGs (*n* = 1–5) 0.1 nM MTXPGs (*n* = 6–7) 0.8 nM	0.1–100 nM MTXPGs (*n* = 1–5) 0.8–100 nM MTXPGs (*n* = 6–7)	>86%	RT 24 h, 7 months −80°C, −20 °C 1 month, five freeze–thaw cycles	[16]
**MTXPGs** (*n* = 3)	RBC pellet (150 µL)	nd.		PPT with 10% TCA	A: H_2_O (10 mM NH_4_HCO_3_) B: ACN gradient; flow: 0.4 mL/min	Acquity HSS T3 1.8 µm; 100 × 2.1 mm	(ESI-LC-MS/MS) MTX-PG_3_ (3) 712. 3 → 308.2	0.238 nM	0.530 nM	1.90 to 500 nM	94.90–114%	nd.	[17]
**MTXPGs** (*n* = 1–7)	RBC pellet (200 µL)	^13^C_5_,^15^N-MTXPGs (1–7)		cold-induced lysis and PPT with perchloric acid	A: H_2_O (10 nM NH_4_Ac; pH = 10) B: MeOH gradient; flow: 0.3 mL/min	Waters XBridge BEH C_18_ 2.5 µm; 100 × 4.6 mm	(ESI-LC-MS/MS) MRM (IS): (1) 455.2 (458.2) → 308.1 (311.1) (2) 584.3 (587.2) → 308.1 (311.1) (3) 713. 3 (716.3) → 308.1 (311.1) (4) 421.7 (423.2) → 175.0 (137.0) (5) 486.2 (487.7) → 175.0 (137.0) (6) 550.7 (552.2) → 175.0 (137.0) (7) 615.2 (616.7) → 175.0 (137.0)	nd.	2.0 nM	2.0–500.0 nM	42.1–100.8%	30 days at −80 °C	[35]
**MTXPGs** (*n* = 3)	VAMS (10 µL)	D_3_-MTXPG_5_		lysis induced by drying, next PPT with 70% perchloric acid	0.1% FA, 0.01% TEA in ACN; isocratic; 1 mL/min	Accucore PFP 2.6 µm; 50 × 2.1 mm	(ESI-LC-MS/MS) MRM (IS): 713. 3 (716.3) → 308.1 (311.1)	nd.	5.0 nM	5–100 nM	>80%	30 days at RT	[36]
**MTXPGs** (*n* = 1–5) and as total	DBS	n.d.		individual MTXPGs lysis induced by drying, next PPT with 70% perchloric acid and extraction with SPE (Oasis Max columns) total MTXPGs lysis of MTXPGs with polyglutamate hydrolase, next as following protocol	A: H_2_O (10 mM NH_4_HCO_3_), pH = 7.50 B: ACN gradient; flow: 0.15 mL/min	Atlantis T3-C_18_ 3 µm; 150 × 2.1 mm	(ESI-LC-MS/MS) MRM (IS): (1) 455.2 → 175.05 (2) 584.3 → 175.05 (3) 713. 3 → 175.05 (4) 842.3 → 175.05 (5) 971.60 → 175.05	nd.	5.0 nM	10–400 nM	44–72%	2 months at −80 °C and RT	[34]
**MTXPGs** (*n* = 1–7)	RBC pellet (200 µL)	D_3_-MTX		cold-induced lysis, and PPT with high temperature	A: H_2_O (10 mM NH_4_HCO_3_ and 5 mM N-HPA, adjusted with FA to 7.50) B: ACN with 5 mM N-HPA gradient; flow: 0.2 mL/min	Synergy Hydro-RP 4 µm; 50 × 1 mm	(ESI-IP-MS/MS) MRM (IS): (1) 455.2 (458.2) → 308.1 (311.1) (2) 584.3 → 308.1 (3) 713. 3→ 308.1 (4) 842.3 → 308.1 (5) 971.3 → 308.1 (6) 1100.4 → 308.1 (7) 1229.4 → 308.1	0.5 nM	1.0 nM	1.0–100.0 nM	51.1– 69.8%	nd.	[38]
**MTX**	plasma (30 µL) DPS (Noviplex^®^) (10 µL)	D_3_-MTX		PPT with MeOH:ACN:H_2_O mixture (40:40:20, *v*/*v*/*v*), For DPS additional extraction following the PPT process with IS solution	A: water with 0.1% FA B: ACN; gradient; flow: 0.7 mL/min	Poroshell 120 SB-C_18_ 2.7 µm; 50 × 4.6 mm	(ESI-LC-MS/MS) 455.2 → 308.2 for MTX 458.2 → 311.20 for IS	nd.	30 ng/mL	30–2000 ng/mL	>92.1%	wet plasma: 3 h RT, 50 days −80 °C DPS: min. one week at RT and 40 °C	[39]
**MTX**	plasma (100 µL)	acetaminophen		PPT with ACN:H_2_O mixture (70:30, *v*/*v*)	A: water with 0.1% FA B: ACN; gradient; flow: 0.4 mL/min	Acclaim 120 C_18 _ 3.0 µm; 50 × 2.1 mm	(ESI-LC-MS/MS) 455.2 → 308.2 for MTX 136.6 → 94.1 for IS	nd.	90 nM	90–1250 nM	40%	24 h at RT	[40]
**MTX;** **7-OH-MTX**	plasma (nd.)	^13^C,D_3_-MTX		nd.	nd.	Avantor Altima HP C_18_-EPS 3 µm; 150 × 2.1 mm	(ESI-LC-MS/MS) 455.0 → 308.0 459.0 → 312.0 for MTX for IS (MTX) 471.0 → 324.0 475.0 → 328.0	nd.	0.02 nM (MTX) 0.16 nM (7-OH-MTX)	nd.	91.5–114.8%	nd.	[41]

ACN—acetonitrile; DAMPA—2,4-diamino-N-10-metylpteroic acid; DBS—dried blood spot; DPS—dried plasma spot; ESI-LC-MS/MS—electrospray ionisation mode in liquid chromatography–tandem mass spectrometry; IS—internal standard; LDMTX—low dose of methotrexate; LLE—liquid–liquid extraction; 7-OH-MTX—7-hydroxy-methotrexate; MTX- methotrexate; MTXPG- methotrexate polyglutamate; RBCs—red blood cells; RT—room temperature; PPT—protein precipitation; SPE—solid phase extraction; TFA—trifluoroacetic acid; VAMS—volumetric–absorptive microsampling.4.4. Post-analytical phase—data interpretation and application for TDM.

Adherence to therapy refers to the degree to which a patient correctly follows medical advice, including taking medication as prescribed, attending scheduled appointments, and making recommended lifestyle changes. High adherence is crucial for achieving optimal health outcomes, especially in chronic conditions like diabetes, hypertension, and mental health disorders. Disease activity score (DAS) is recommended by EULAR (European League Against Rheumatologism) for treatment response assessment—not only with MTX [7]. This scale uses the number of tender and swollen joint episodes, holistic patient assessment, and biochemical inflammation biomarkers (including RBC sedimentation rate) to qualify the patient as suitable, moderate, or non-responder, including prognostic clinical remission in some cases [17]. The data discussed demonstrate a positive correlation between DAS and MTXPG levels in whole blood, especially for MTXPG_3_. It should be noted that PG determination should be initiated after one month from therapy for appropriate synthesis of MTXPG_3_, which is predominated in RBCs. Non-adherence in RA evaluated using a compliance questionnaire in the Marras et al. study ranged from 59% to 107%, which corresponds to under- and overuse during the observed period of pharmacotherapy [42]. Adherence is highly variable due to various definitions of adherence and cut-off levels implemented in the analysed studies [9,10,11,12,13,14].

MTXPGs are associated with the toxicity and side effects of MTX administration, which is also detailed in LDMTX protocol. The clinical response for MTX in RA patients is highly variable, ranging from 30 to 60%. Notably, 95% of administered MTX is metabolised within 24 h. Therefore, MTX levels do not fully represent an agreement between treatment and exposure.

MTXPG_1-2_ was detected in plasma in small concentrations, while MTXPG_1-5_ was detected in all matrices except plasma, and MTXPG_6-7_ is not detected in any matrix under LDMTX protocol [21,22,23]. Similarly, van Boer et al. concluded that the sixth and seventh MTXPGs are not detectable in RBCs, and the mean individual number of detected PGs is increasing as follows: MTXPG_5_, _−2_, _−4_, _−1_, _−3_ [25]. This finding was concluded based on an analysis of the RA patient group under 50–121 days of treatment with an MTX dose of 10–25 mg per week.

The Mo et al. study concluded that there are interracial differences in gene polymorphism, which caused differences in MTXPG synthesis in cells (Caucasian versus Chinese populations) [33]. Gene polymorphism may cause differences in therapeutic efficacy—according to MTXPG production in RBCs, this is caused by mutations in RBC penetration. In that study, concentrations of MTXPG were 10.1 ng/mL (0.4–59.4 ng/mL), expressed as total PGs. The analysed population included RA adult patients under LDMTX protocol (10–15 mg, iv. weekly dose) [33].

Bluett et al. deducted that urine may be a beneficial matrix for MTX and 7-OH-MTX determination and adherence assessment [32]. MTX can be detected in urine even 4 days after drug administration, which is more favourable compared to blood. 7-OH-MTX levels (>LLOQ) are detectable in samples collected after 46 h of oral drug administration. Despite the above, the MTX/MTX-7-OH ratio seems to be a candidate for the PK/PD monitoring of MTX, especially in LDMTX [14,32].

The crucial finding about the additional monitoring of biochemical parameters during LDMTX was concluded in the Sonemoto et al. study [18]. A combination of NSAIDs (non-steroidal anti-inflammatory drugs) and MTX increased ALT (Alanine Aminotransferase) and AST (Aspartate Aminotransferase) levels. The study included rheumatic outpatients (two males, seven females; 52–80 years old) with 8 or 10 mg of MTX weekly. MTX concentration ranged from 297.8 to 1164.1 nM [18].

Huerta-Garcia performed a study with elements of population modelling of 1- and 2-compartmental analysis, including PK/PD estimation [17]. In the study, 89 women with RA were included. In total, 41.6% of them were obese, 30.3% were overweight, 24.7% were average weight, and 3.4% were underweight according to BMI. Compartmental analysis was performed according to first-order absorption and elimination. The validated pharmacokinetic model has been set as follows to estimate PK parameters. The mean population volume of distribution has been calculated as V_d_ = 52.4 L, with an initial dose range of 7.5–17.5 mg. MTXPG_3_ has been related to BMI, cell number, dose and time under treatment with MTX, and cells/µL; additionally, higher MTXPG_3_ levels positively correlated with response to the therapy [17]. According to MTXPG_3_, similar findings have been found in the Brady et al. study [36].

Non-adherence is also associated with children, who are a high-risk group. According to time and dose amount, Möhlmann defined adherence in 43 paediatric patients with Juvenile Idiopathic Arthritis (JIA) as a plasma concentration of MTX above the threshold limit [41]. Additionally, 7-OH-MTX has been applied as a surrogate of adherence, and the JAMAR (Juvenile Arthritis Multidimensional Assessment Report) questionnaire has been used for clinical evaluation. The adherence rate to weekly therapy with MTX was 88% after initiation and 77% after one year of treatment [41]. The half-life time of MTX is short. Therefore, adherence may be the effect of white-coat compliance. The ideal method for adherence control is introducing a home-based self-sampling process for more frequent MTX or MTXPG level determination as biomarkers of drug intake adherence in patients [41].

Hawwa et al., in two studies, evaluated MTXPGs analytically and clinically as potential biomarkers of non-adherence in a paediatric population with rheumatic disorders. The team concluded that for adherence improvement, the subcutaneous drug form could be considered an alternative to oral tablets with MTX [13,34].

TDM of MTX and its metabolites is also used in treatments for inflammatory diseases other than RA. For example, Meeberg et al. presented exciting work about TDM during pharmacotherapy in patients with Crohn’s disease [43]. They concluded that during MTX treatment, MTXPG3 concentration is correlated with better drug survival and lower faecal calprotectin levels (a biomarker to detect intestinal inflammation) [43,44].

Some studies compared different methods of sample collection or MTX determination. Interestingly, Cao et al. proved that MTX levels determined in DPS Noviplex^TM^ (currently Telimmune^TM^) cards and wet plasma are statistically comparable [39]. Therefore, the mentioned methods may be used interchangeably for the sampling process. In another experiment, the LC-MS/MS methodology was compared with immunoassay. However, we noted the characteristics of the potential interference for IAs in paragraph 3. Still, Opitz et al. and Bougié et al. demonstrated a high correlation between paired results obtained for determining MTX using LC-MS/MS and IA techniques [29,31].

## 5. The Summary of Best Practises in TDM Process Implementation Clinically in the LDMTX Protocols

The laboratory test for MTXPGs is commercially available and used in current clinical practice. But, on the other hand, there are a few confirmed implementations, mainly in the United States [45,46,47,48]. Therefore, based on our own experience and a comprehensive literature review, we proposed a list of potential clinical problems of TDM during LDMTX protocol treatment application.

The crucial underachievement of LDMTX pharmacotherapy may be associated with the following [8,9,10,11,12,13,14,15,16,17,18,19,20,21,22,23,24,25,26,27,28,29,30,31,32,33,34,35,36,37,38,39,40]:

1. Toxicity (hipermethotrexatemia) or underexposure (hipomethotrexatemia):
  - Check the basic biochemistry parameters, i.e., ALT, ASP, and renal function biomarkers.
  - Check potential interaction with other drugs concomitantly used during therapy with MTX (interactions drug–drug) according to pharmacokinetics and pharmacodynamics.
  - Check MTX levels (or metabolites).
    ✓ Samples for MTX determination must be collected at appropriate time points—max. 24 h after drug administration due to rapid MTX metabolism (the results should be interpreted according to time after drug intake).
    ✓ Serum/plasma samples are suitable for MTX monitoring (eventually 7- OH-MTX). MTX monitoring during LDMTX treatment may be beneficial in poisoning with MTX.
    ✓ Whole blood (especially RBC pellets) is the suitable matrix for MTXPG determination.
    ✓ In the case of metabolite monitoring, the following is recommended:
      ▪ Consider urine as a matrix for 7-OH-MTX determination;
      ▪ MTXPG determination may be considered a biomarker of long-term exposure to MTX.
  - Check the potential interaction associated with individual patient factors (e.g., age, ethnicity, sex, co-morbidities).
  - Check the correctness of drug intake by the patient.
2. Various tools and protocols may identify non-adherence to the therapy [40,41,42]:
  - Metabolite concentration monitoring
    ✓ MTXPGs are surrogates of MTX exposure even over 90 days (life-time of RBCs), and is considered a long-time period adherence biomarker:
      ▪ MTXPG concentrations may be expressed as total (sum of MTX after hydrolysis *and* ‘*free*’ *MT*X) or individually per MTXPG.
      ▪ MTXPG concentrations should also be interpreted holistically with other biochemical parameters and drug doses.
    ✓ 7-OH-MTX may be considered a short-term adherence biomarker in serum/plasma or urine.
    ✓ Consider the application of the remote home-based microsampling technique as a unique tool for drug/metabolite monitoring.
  - Consider implementing other tools for adherence to the therapy control during pharmacotherapy, such as drug registration, the patient’s diary, retrospective questionnaires, tablet count, clinical scores, and interviews.

## 6. Conclusions

The clinical monitoring of pharmacotherapy using both LDMTX and HDMTX requires special attention according to drug exposure, toxicity, side effects, adherence monitoring, and, finally, dosing adjustment according to the introduction of rescue therapy. In an ‘ideal world’, the analytical method should assume a relatively wide calibration range (including estimated MTX/metabolite concentration independent of therapeutic protocol). Additionally, selectivity and specificity are crucial when metabolites are also monitored. The LC-MS/MS platforms are still recognised as the gold standard for the determination of MTX and its metabolites, especially in low-dosing protocols during non-adherent episodes. Due to the insufficient sensitivity of immunochemical assays or chromatographic methods with ‘more classic’ detection methods (i.e., UV or FLD), LC-MS/MS platforms are the methodologies of choice. On the other hand, there are few gaps in the characteristics of LC-MS/MS, such as the expensive costs of analytical platforms, appropriate training personnel, and chemical purity. Therefore, cost-effective alternatives to LC-MS/MS for routine clinical use is strictly needed.

The proper education of patients, according to the drug administration schedule, potential side effects, and benefits of treatment, should be carefully explained to each patient on every control visit. MTXPG levels may be helpful for dose adjustment, toxicity detection, and adherence evaluation. However, the monitoring of MTX or their metabolite concentration are not strictly required, but, as we have demonstrated in the presented research, is crucial and beneficial, not only in RA, but also in other autoimmunological diseases when MTX is administered.

To sum up, the implementation of routine MTXPG measurement in daily clinical practice could be useful for monitoring the effects of pharmacotherapy with MTX. It would also help in selecting the appropriate drug dose, namely increasing it if there is no therapeutic effect or reducing it if side effects occur.

## Figures and Tables

**Table 1:** Essential pharmacokinetics parameter characteristics for MTX [1,3,4,15].

Pharmacokinetics Variable	Mean Value
bound with proteins [%] (unbound fraction of drug)	50 (0.5)
half time—t_1/2_ [h] adult child	8–10 5–9
time to steady state [h] adult child	24–48 24–48
excretion in urine [%]	40–50 (LDMTX) 1–20 (7-OH-MTX) unknown DAMPA
oral dose bioavailability [%]	50–100 (70–80% after one week under 15 mg MTX dose exposure)
volume of distribution [L/kg]	0.75
time to peak concentration (t_max_) [h]	1–4
maximal concentration (C_max_) [µM]	0.25–2.0
toxic concentrations [µM]	>0.02 (1–2 weeks under LDMTX)

DAMPA—2,4-diamino-N-10-metylpteroic acid; LDMTX—low dose of methotrexate; 7-OH-MTX—7-hydroxy-methotrexate.

## Data Availability

Not applicable.
